# Diseases of the Kidneys

**Published:** 1905-02-04

**Authors:** 


### DISEASES OF THE KIDNEYS.

(*Continued from page* 319.)

**Nephritis in the Young.**—Arthur Hall[2] describes a case of interstitial nephritis in a girl aged eight, with attacks of convulsions and hæmorrhages from the stomach, lungs, nose, ears, and under the skin, as well as optic atrophy. The kidneys were atrophied and fibrous, with the cortex much diminished. Cardiac hypertrophy, pleurisy, pericarditis also occurred before death. Guthrie has recorded seven fatal cases in children, and, as West says, this form of the disease is probably often overlooked; but still instances are certainly rare. *Cyclical* or functional albuminuria is thought by H. G. Armstrong[3] to occur in from 12 to 15 per cent. of schoolboys, more commonly perhaps in the summer than in the winter. One important symptom consists in fainting attacks when the boy is standing up in chapel or at drill. The pulse tension in such cases is low, the heart dilated and very variable in its action, and the family history neurotic. The main diagnostic points from true Bright's disease are the absence of casts, the high specific gravity of the urine, and the disappearance of albumen and palpitation when the patient is kept in bed, or at least its occurrence only after first getting up in the morning. The prognosis is good; the patient should not be coddled or made more nervous. Active outdoor exercise, except perhaps long-distance races, is advisable. The condition usually ceases of itself before 25, and the writer thinks that before this age an uncomplicated attack should be no bar to insurance at ordinary rates. If persistent after 25 the prognosis is worse. An astonishing thing about this complaint is the frequency with which it is overlooked, and boys sent to school with letters from medical men stating that they are unfit for sports and outdoor exercise, etc., owing to some disorder of the heart or digestion, the condition of the urine being quite unnoticed. The fainting attacks, palpitation, and general "slackness" are usually all that are complained of in these cases. Much discussion has taken place as to the effect on infants of food preservatives, such as borax. Milk sometimes contains 15 grains of borax to the pint, butter 40 grains to the pound, beef and ham 250 grains to the pound, and the use of it seems to be increasing, as it does not withdraw water from the substance treated, and is, moreover, a good preservative. As to the vexed question whether borax may produce or aggravate disease of the kidneys, Harrington[4] has carried out some experiments on cats, and found that five out of six fed with borated food showed marked kidney lesions. It is certain that very large amounts of these preserved foods must be eaten to give as much borax as these animals received, but the fact that borax can at all produce kidney lesions is important. Harrington,[5] moreover, finds borax almost universal in canned foods, milk, and butter. Thus it exceeded more than 1 per cent. in every one of 51 specimens of American pressed beef. He adds that the borax once applied to meat cannot be removed from the tissues either by soaking or boiling. Hale White[6] has an interesting paper on chronic Bright's disease in the young, and emphasises the rarity of its origin from the acute disease. However, such cases do arise, and some few from scarlatinal nephritis; but true tubal nephritis (as distinct from mere albuminuria) only occurs in 2·8 per cent. of attacks of scarlatina; and of these very few become chronic, so that the number of chronic cases following scarlatina is very small. Some other cases have started, not from an acute form, but from a granular kidney, rare though that is in the young. The large white kidney may of course follow after acute nephritis. He also points out that the younger the patient is who has chronic renal disease the more rapidly does the fatal termination come to pass, and that the mode of death is always by uræmia. It is important to distinguish two forms of retinitis seen in renal cases, since in the degenerative form death may be foretold within a short period. Here the vessels are thick, the hæmorrhages are numerous, the white patches are glistening, the disc is not swollen nor the retina hazy. On the other hand the inflammatory form has no such fatal augury, but here the hæmorrhages are few, the white patches are opaque, the disc swollen, and the retina cloudy. Rose Bradford[7] brings forward many interesting points on the distinction of different forms of Bright's disease. He discusses the question of an internal secretion by the kidney, and concludes that the kidney has no group of cells which are adapted for that purpose, and that there is no conclusive evidence in favour of such a secretion, though the wasting and the great increase of urea after removal of more than two-thirds of the kidney could be best explained on that supposition. The poisons which can cause renal lesions are numerous. First there are the heavy metals, such as lead, and vegetable poisons such as abrin; animal ones, such as cantharidin and the blood of eels; the products of bacterial action, and certain poisons derived from cells of the body. It is furthermore possible to classify them, as they act on the glomeruli or on the epithelium of the convoluted tubules, but in none of these do we get one symptom which is fairly common in true Bright's disease, viz., dropsy. Much discussion has arisen as to whether we should distinguish acute nephritis from acute Bright's disease, and here, too, the main distinction seems to be that dropsy occurs frequently in the latter and not in the former. It is not that acute Bright's disease is necessarily a more severe form of acute nephritis. Dropsy may occur in very mild attacks, and, on the other hand, it is almost entirely absent in suppression of urine and in cystic kidney. It would seem that in acute nephritis there is a poison which affects the kidney only, and in Bright's disease one which also affects the blood-vessels generally. Among types of acute nephritis there is the group which occurs in febrile diseases. The affection is mild and transitory, and the lesions in the kidney are scattered widely apart, but there is a possibility that this febrile albuminuria may be the origin of many serious cases of chronic Bright's disease, and perhaps of functional albuminuria. *Syphilis* is often an unsuspected cause of nephritis, and this type is marked by a very large amount of albumen, which does not lessen under a milk diet. Little or no blood is present, and few casts. The urine is not much diminished, dropsy is slight or absent, but severe uræmia may develop. In spite of theoretical objections, mercury and other antisyphilitic remedies should be given. The affection occurs during the secondary stage, and must be distinguished from amyloid disease, which is seen in the tertiary period. One type of chronic Bright's disease is often overlooked—viz. that connected with *the small white kidney*. It occurs in young people often under 25 years of age. It is often latent until a rapidly fatal uræmia sets in. There is no dropsy, the urine is abnormally copious, the albumen is very plentiful, but the casts are less numerous than in the large white kidney, where dropsy is so marked. The writer does not think that these cases are necessarily later stages of the large white kidney, or due to red granular kidneys in which acute attacks have come on. Generally they arise independently, though they sometimes may follow after an acute affection. The vascular and cardiac changes which are so marked in the red granular kidney may be almost entirely absent in the small white form. In this latter type, too, are found the most severe and rapidly fatal attacks of uræmia, sometimes maniacal, sometimes dyspnœic, but nearly always quickly fatal.

[2] Lancet, July 23. [3] Brit. Med. Jour., Oct. 8. [4] Ibid., Sept. 17. [5] Lancet, Oct. 1. [6] Clin. Jour., June 15. [7] Lancet, July 16.

